# Editorial to “Atypical atrial resetting with ventricular extrastimulus during tachycardia: What is the mechanism?”

**DOI:** 10.1002/joa3.13144

**Published:** 2024-09-02

**Authors:** Tatsuya Hayashi, Hideo Fujita

**Affiliations:** ^1^ Division of Cardiovascular Medicine Saitama Medical Center, Jichi Medical University Saitama Japan

**Keywords:** nodoventricular pathway, paroxysmal supra ventricular tachycardia, reset

Editorial to “Atypical atrial resetting with ventricular extrastimulus during tachycardia: What is the mechanism?”^1^

The use of catheter ablation to treat tachyarrhythmias initially involved targeting the atrioventricular node (AV node). Over time, as specific arrhythmic circuits have been discovered, it has become feasible to perform ablations at safer and more efficient locations beyond the AV node. This technique is now utilized to address a wide range of arrhythmias. Supraventricular tachycardia (SVT) is generally classified into atrioventricular nodal reentrant tachycardia (AVNRT), orthodromic reciprocating tachycardia (ORT), and atrial tachycardia (AT), all of which have been successfully treated with catheter ablation, and SVT treatment is now largely established.^2^ However, despite these successes already achieved, recent electrophysiological study (EPS) advancements have uncovered new insights into SVT, suggesting that the success of catheter ablation and understanding the true mechanism of the tachycardia circuit are different things. This is exemplified by the recent “re‐discovery” of the nodoventricular (NVP) and nodofascicular pathways (NFP), which can often exist as a bystander pathway in successful AVNRT cases with slow pathway ablation.^3^ These evolving insights have added complexity to SVT differentiation, necessitating a broader differential diagnosis approach during treatment. We electrophysiologists have witnessed the maturation of diagnosis and treatment of SVT over decades, and now we realize that it is further developing into something like “SVT ‐Season 2.” In this study, Kobari et al. reported a complex case of supraventricular tachycardia that required detailed analysis for diagnosis and was successfully ablated.^1^ In this case, the first EPS findings suggested that the mechanism of SVT was ORT. For example, the initial atrial (A) and ventricular (V) activation at the time of overdrive ventricular stimulation cessation was a V‐A‐V sequence, and the postpacing interval at the stimulation site minus the tachycardia cycle length was 67 ms. The treatment approach for “pre‐modern” SVT may involve directly ablating the earliest atrial excited site without further detailed examination. However, this approach is insufficient in the current “SVT‐season 2,” and a deeper understanding is essential. It is crucial to distinguish whether the diagnosis is ORT via a slow conduction accessory pathway (AP) or through an NVP. In ORT involving a typical or slow conduction AP, the atrium is an essential part of the tachycardia circuit, whereas in ORT using an NVP, the atrium is not involved in the tachycardia circuit. Based on this background, this paper determined that the atrium is not a crucial part of the tachycardia circuit as the AH duration during tachycardia is significantly different from the AH duration during atrial stimulation of the same tachycardia cycle length,^4^ identifying the presence of NVP. This method is crucial for differentiating complex tachycardia, especially when excluding tachyarrhythmia, which includes atria into essential circuits such as AT or ORT using a usual atrioventricular AP. When performing this differentiation procedure, it is necessary to ensure that dual atrioventricular conduction does not alter the AH duration.

In addition to this discussion, the key EPS finding, in this case, was the atrial preexcitation with a single premature ventricular stimulus delivered during ongoing tachycardia at the time of His bundle (HB) refractoriness, without producing morphological changes in the ventricular activation sequences at the coronary sinus (CS). This finding indicates that APs are present in ventriculoatrial conduction and that extraventricular stimulation is conducted to the atria without capturing the ventricle at the contralateral site of the earliest atrial activation near the mitral annulus, suggesting that this ventricular location is not an essential part of the tachycardia circuit. This observation is only possible if the ventricular insertion of the AP is located at a more apical site of the ventricle, and the length of the accessory pathway can be longer than the “usual” atrioventricular AP. Recent literature highlights “paradoxical reset,” where a single premature ventricular stimulus during the HB refractoriness prolongs the atrial cycle length rather than shortening it. This indicates the presence of concealed NVP, even though it may not contribute to the tachycardic circuit (even though it may be a bystander).^5^ In this case, “atypical reset” means that the ventricular activation sequence of CS during the reset matched the ventricular activation sequence during the tachycardia, offering a new perspective. This “atypical reset” finding is important as it provides insight into the location of ventricular insertion of the AP, which can change the ablation strategy. In other words, this paper highlights the importance of closely observing the ventricular activation sequence in the CS during the reset phenomenon. Future studies should investigate whether this atypical resetting is specific to ORT with NVP or if it can also occur in typical ORT with long APs.

Furthermore, it should be noted that in this case, the ventriculoatrial conduction during parahisian pacing exhibited an apparent AV nodal pattern, not an AP pattern. If the conduction via the AV node is sufficiently faster than the conduction via the AP, the conduction via the AP can be masked.

This case allows for a diagnosis using all the latest EPS knowledge. The “SVT‐season 2” should continue gaining momentum, and we must keep ourselves updated.

## CONFLICT OF INTEREST STATEMENT

Authors declare no conflict of interests for this article.

## PERMISSION TO REPRODUCE MATERIAL FROM OTHER SOURCES

Yes.
